# Protected Time for Electronic Health Record Work and Physician Productivity

**DOI:** 10.1001/jamanetworkopen.2025.46550

**Published:** 2025-12-02

**Authors:** Naga S. Kanaparthy, A. Jay Holmgren, Yu Sun, Hyung Paek, Brian Williams, Robert McLean, Robert Doolan, Katherine E. Goodman, Lisa Rotenstein, Edward R. Melnick

**Affiliations:** 1 Department of Emergency Medicine, Yale School of Medicine, New Haven, Connecticut; 2 Department of Biomedical Informatics and Data Science, Yale School of Medicine, New Haven, Connecticut; 3 VA Connecticut Healthcare System, US Department of Veterans Affairs, West Haven, Connecticut; 4 Department of Medicine, University of California, San Francisco; 5 Department of Health Informatics, Yale Graduate School of Art and Science, New Haven, Connecticut; 6 Department of Medicine, Division of General Internal Medicine, University of Colorado School of Medicine, Aurora; 7 Department of Epidemiology and Public Health, University of Maryland School of Medicine, Baltimore; 8 University of Maryland Institute for Health Computing, North Bethesda; 9 Department of Internal Medicine, Yale School of Medicine, New Haven, Connecticut

## Abstract

This cohort study evaluates whether reserving 1 appointment slot per half-day for internal medicine physicians to complete asynchronous, electronic health record (EHR)–based work is associated with changes in productivity, EHR use, and patient communication volume.

## Introduction

Electronic health record (EHR)–based patient portals have enabled asynchronous communication between patients and physicians^1,2^ but have contributed to fragmented and inefficient work and subsequent physician burnout.^3,4^ In response, a large ambulatory network in the Western US set aside 1 appointment slot per half-day for physicians to complete asynchronous EHR-based tasks, such as messaging, prior authorizations, and prescription refills, at the physician’s discretion. We assessed changes in productivity and EHR use associated with this intervention relative to a control practice network.

## Methods

In this cohort study, we conducted a comparative interrupted time series analysis (CITSA), a quasi-experimental method comparing outcome level and trend changes over time between groups. We studied general internal medicine physicians from 2 ambulatory practice networks between November 2021 and June 2024. All eligible physicians were included. Beginning in November 2022 and continuing onward, the intervention network reserved 1 appointment slot per half-day (20-30 min/slot) for asynchronous work, while the control network continued usual care. Both groups operated primarily under fee-for-service models with limited value-based incentives.

Work relative value units (RVUs) were obtained from each site’s administrative databases to assess productivity. For clinicians’ EHR activity, we used deidentified log data from Epic Signal (Epic Systems). Log data have been used previously to evaluate EHR use and clinician workload.^5^ EHR after-hours use, EHR nonworkday use, and patient messages (eTable in Supplement 1) were selected as proxy metrics for physician well-being.^4^ All data were analyzed at the physician-week level as continuous measures; no imputation was required. A CITSA was performed using Stata/SE version 15.1 (StataCorp) to assess immediate changes and postintervention trend changes. We adjusted for physician age, gender, and panel size, and SEs were clustered at the physician level to account for intraphysician correlation. A contemporaneous control network was included to strengthen causal inference, as CITSA models estimate group-specific levels and slopes, mitigating bias from systemwide time-varying confounders impacting both systems.^6^ Statistical significance was defined as *P* < .05. The institutional review boards of Northeast Medical Group (control site) and the University of Colorado (intervention site) approved this study with a waiver of informed consent because this was a secondary analysis of deidentified data.

## Results

The control and intervention groups included 89 and 41 physicians, respectively, with complete follow-up (Table). Following the intervention, mean RVUs declined slightly in the intervention network (48.7 to 45.1) but remained stable in the control practice. EHR after-hours use, EHR nonworkday use, and patient messages decreased in both networks.

**Table.** Physician, Practice, and EHR Metrics by Site at Baseline and Over Time

Characteristic	Mean (SD)		
	Control group	Intervention group	Total
Practice characteristics			
Panel size	1380.6 (494.9)	608.1 (387.7)	1127.2 (587.3)
Patient age, y	61.3 (4.3)	60.9 (6.9)	61.2 (5.2)
Physician FTE	0.89 (0.20)	0.39(0.26)	0.74(0.32)
No. of practice sites	51	5	56
Physician characteristics			
No. of physicians	89	41	130
Physician age, No. (%)			
<35 y	4 (4.5)	0	4 (3.1)
35-44 y	16 (18.0)	8 (19.5)	24 (18.5)
45-54 y	27 (30.3)	19 (46.3)	46 (35.4)
55-64 y	25 (28.1)	10 (24.4)	35 (26.9)
≥65 y	17 (19.1)	4 (9.8)	21 (16.2)
Physician sex, No. (%)			
Female	43 (48.3)	27 (65.8)	70 (53.8)
Male	46 (51.7)	14 (34.2)	60 (46.2)
Outcome metrics			
Scheduled h/wk	24.4 (7.5)	9.7 (6.2)	19.8 (9.9)
Preintervention			
Relative value units/wk	116.0 (46.9)	48.7 (31.4)	94.8 (52.8)
EHR after-hours use, min/wk	186.4 (184.6)	153.1 (123.0)	176.6 (169.5)
EHR nonworkday use, min/wk	104.5 (118.9)	121.9 (78.2)	109.9 (108.3)
Patient messages/wk	27.3 (23.5)	33.1 (33.0)	29.1 (27.0)
Postintervention			
Relative value units/wk	116.4 (47.0)	45.1 (28.8)	93.9 (53.6)
EHR after-hours use, min/wk	170.9 (165.1)	125.8 (100.5)	157.6 (150.3)
EHR non-workday use, min/wk	103.2 (124.9)	108.5 (76.0)	104.9 (111.7)
Patient messages/wk	25.8 (22.4)	27.3 (17.4)	26.3 (20.9)

Abbreviations: EHR, electronic health record; FTE, full-time equivalent.

In the CITSA, the intervention was associated with an immediate relative reduction of 13.29 RVUs/wk (*P* < .001) compared with the control group, followed by a postintervention trend increase of 0.43 RVUs/wk (*P* = .002) compared with the control group (Figure). After-hours and nonworkday EHR use declined immediately by 24.78 min/wk (*P* = .007) and 28.57 min/wk (*P* = .002), respectively, with no postintervention relative trend changes. Patient messages increased by 3.39 messages/wk (*P* = .008) at intervention, followed by a relative postintervention decline of 0.32 messages/week (*P* < .001). Full preintervention and postintervention trends are reported in the Figure.

**Figure.**
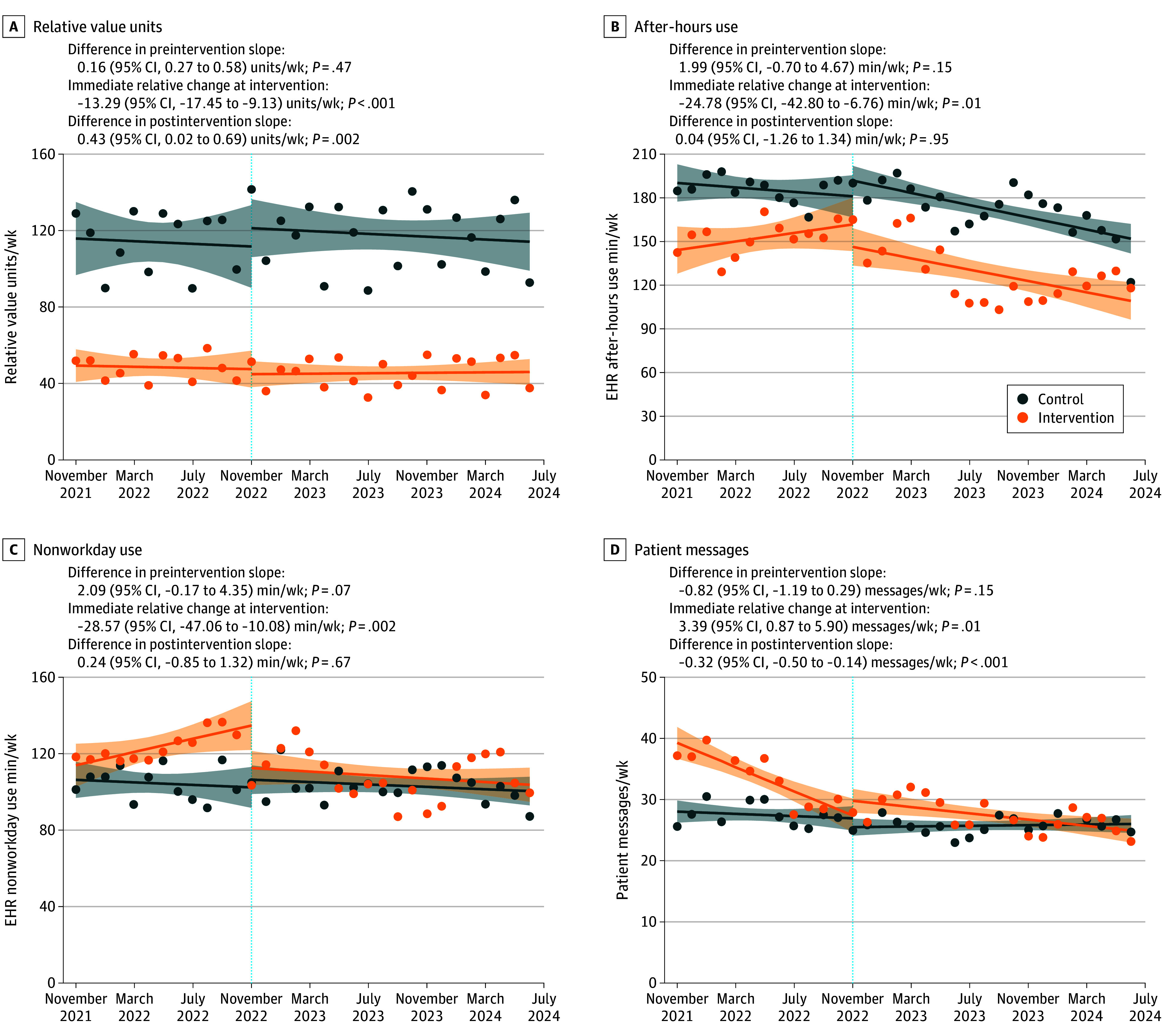
Comparative Interrupted Time Series Analysis of Appointment Slot Intervention Comparative interrupted time series analysis showing the outcomes associated with implementing 1 dedicated appointment slot per half-day session in November 2022 (dotted vertical line, marking the start of the first week of the intervention period) on relative value units per week, electronic health record (EHR) after-hour use per week, EHR nonworkday use per week, and patient messages per week. Changes reflect differences in both level and trend over time relative to the control group following the intervention. Orange circles and lines represent observed and projected values for the intervention group, while blue circles and lines denote those for the control group. Shaded bands around each line indicate 95% CIs for the projected value. Estimates reflect relative differences between the intervention and control groups, distinguishing immediate level changes at the time of the intervention from postintervention trend differences, as modeled in the controlled interrupted time series analysis. Panel annotations indicate estimated preintervention, immediate, and postintervention slopes. The decline in patient message volume in the intervention group before implementation (bottom right panel) likely reflects preexisting institutional efforts to manage inbox burden—initiatives that, while separate from the intervention studied here, served as a lead-in to its formal launch.

## Discussion

In this cohort study with a CITSA, we found that reserving 1 appointment slot per half-day for EHR work reduced after-hours and nonworkday EHR use, with only a small decrease in productivity measured by RVUs, suggesting that providing dedicated time for asynchronous work may alleviate clinician burnout without compromising revenue. Indeed, a pre-post survey at the intervention site found that physician burnout was 81% lower after the intervention. Limitations include comparing ambulatory networks with differing practice sizes and the more academic, lower clinical full-time equivalent (FTE) profile of the intervention group, limiting generalizability. These findings may not apply to non–fee-for-service or nonacademic settings, where productivity metrics and clinical FTE expectations differ. Month-year fixed effects and a concurrent control group help address temporal trends; however, the lack of archived data before November 2021 limits our ability to further control for additional seasonality impacting only one group. Despite structural differences, retaining a control group improves validity by accounting for secular trends and concurrent institutional initiatives.^6^ Future studies could directly measure burnout metrics.

## Notes

Supplement 1. **eTable. **Mapping of Metrics Used in This Article to Corresponding EHR Signal Metrics

Supplement 2.
Data Sharing Statement
