# Supplementary material for: Protected Time for Electronic Health Record Work and Physician Productivity
Source: JAMA Netw Open. 2025 Dec 2;8(12):e2546550. doi: 10.1001/jamanetworkopen.2025.46550 (PMC12673411; doi:10.1001/jamanetworkopen.2025.46550)
Supplement: Supplement 1. — eTable. Mapping of Metrics Used in This Article to Corresponding EHR Signal Metrics [file jamanetwopen-e2546550-s001.pdf]

Supplemental Online Content

Kanaparthy NG, Holmgren AJ, Sun Y, et al. Protected time for electronic health record work and physician productivity. *JAMA Netw Open*. 2025;8(12):e2546550.
doi:10.1001/jamanetworkopen.2025.46550

eTable. Mapping of Metrics Used in This Article to Corresponding EHR Signal Metrics

This supplemental material has been provided by the authors to give readers additional information about their work.

© 2025 Kanaparthy NG et al. *JAMA Network Open*.

eTable. Mapping of Metrics Used in This Article to Corresponding EHR Signal Metrics

**Corresponding Metric in manuscript**	**Metric from Epic Signal (Epic Systems, Verona)**
Electronic Health Record (EHR) After-hours use	EHR Time Outside Scheduled Hours
Electronic Health Record (EHR) Non-Workday use	EHR Time on Unscheduled Days
Patient Messages	Patient Medical Advice Requested Only (PMAR) - In Basket Messages Received

Abbreviations: EHR = Electronic Health Record; PMAR = Patient Medical Advice Request. In Basket refers to Epic's internal electronic messaging system

© 2025 Kanaparthy NG et al. *JAMA Network Open*.
