# Supplementary material for: Protected Time for Electronic Health Record Work and Physician Productivity
Source: JAMA Netw Open. 2025 Dec 2;8(12):e2546550. doi: 10.1001/jamanetworkopen.2025.46550 (PMC12673411; doi:10.1001/jamanetworkopen.2025.46550)
Supplement: Supplement 2. — Data Sharing Statement [file jamanetwopen-e2546550-s002.pdf]

Data Sharing Statement

Kanaparthy. Protected Time for Electronic Health Record Work and Physician Productivity. *JAMA Netw Open*. Published December 02, 2025. doi:10.1001/jamanetworkopen.2025.46550

Data
Data available: No
